# Pediatric Behavioral Health during the COVID-19 Pandemic: Expert Advice for Preparedness, Response, and Recovery

**DOI:** 10.3390/ijerph20115920

**Published:** 2023-05-23

**Authors:** Kimberly Burkhart, Carolyn E. Ievers-Landis

**Affiliations:** 1 Department of Psychiatry, University Hospitals Cleveland Medical Center, Case Western Reserve University School of Medicine, Cleveland, OH 44106, USA; 2 Department of Pediatrics, Rainbow Babies & Children’s Hospital, University Hospitals Cleveland Medical Center, Case Western Reserve University School of Medicine, Cleveland, OH 44106, USA; carolyn.landis@uhhospitals.org

**Keywords:** commentary, pediatric behavioral health, disaster preparedness, COVID-19, pandemic, disaster response, disaster recovery, workforce development

## Abstract

The COVID-19 pandemic exacerbated the child mental health crisis and existing disparities. Child anxiety, depression, suicide attempts and completions, and mental-health-related emergency department visits significantly increased. In response to this crisis, the Administration for Strategic Preparedness and Response (ASPR) developed behavioral health task forces associated with funded pediatric centers of disaster excellence. The Health Resources and Services Administration (HRSA) funded the Pediatric Pandemic Network (PPN) to prepare for future endemics and pandemics, with behavioral health identified as a priority in mitigation, preparedness, response, and recovery. This commentary provides insights from pediatric disaster preparedness and response behavioral health subject matter experts. Our roles have been to identify how to build behavioral health professional competencies across disciplines and various medical settings and to strengthen emergency interdisciplinary behavioral health care capability regionally and at the national level. Specific examples of interdisciplinary training and demonstration projects are included as models for enhancing behavioral health situational awareness and developing curricula to support preparedness and response for the current ongoing pandemic and future natural and biological disasters. This commentary also includes a call to action for workforce development to move beyond a boots-on-the-ground mentality for pediatric behavioral health disaster preparedness and response toward a more inclusive role for behavioral health providers of varied specialties. This means that behavioral health providers should become more informed of federal programs in this area, seek further training, and find innovative ways to collaborate with their medical colleagues and community partners.

## 1. Introduction to Phases of the Disaster Cycle

**Figure 1:**
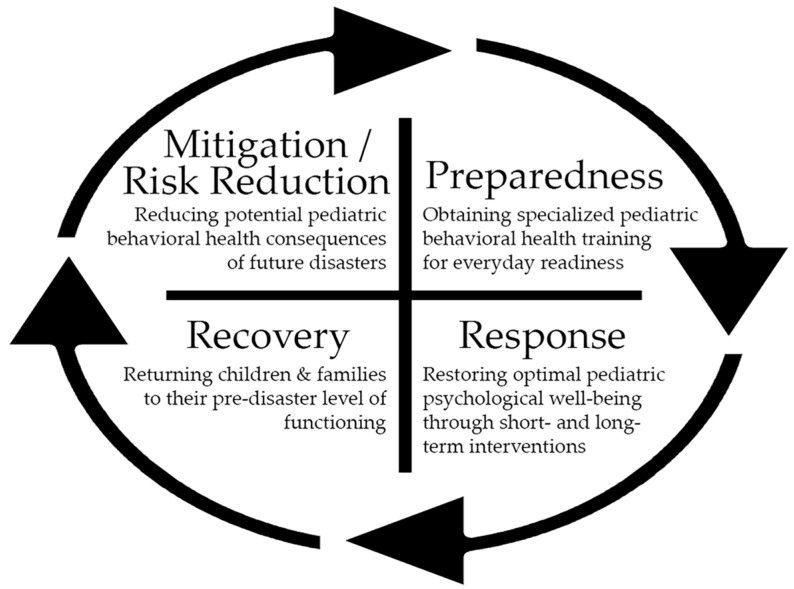
The disaster management cycle for pediatric behavioral health: mitigation/risk reduction, preparedness, response, and recovery.

Disasters fall into the categories of natural or manmade, with pandemics/endemics being one example of a natural disaster. Professionals in emergency management have identified five phases of the disaster cycle as prevention, mitigation, preparedness, response, and recovery. All phases should include a behavioral-health-informed approach with special consideration given to pediatrics because children are the most vulnerable when a disaster occurs. In the prevention phase, potential threats/risks and sources of community resiliency are identified that could serve to minimize the psychological impact of a disaster on children and their families. The mitigation phase involves taking steps to prevent and reduce the cause, impact, and potential consequences of future disasters. The preparedness phase consists of ongoing specialized behavioral health training with evaluation and quality improvement to ensure everyday readiness. In the response phase, the focus is on restoring optimal psychological well-being and functioning through both short- and long-term behavioral health interventions. The recovery phase involves stabilizing and restoring all behavioral health supports, returning children and their families to their pre-disaster level of functioning, and/or creating a new normal [1]. See Figure 1 for a graphical representation of the disaster management cycle for pediatric behavioral health.

Considering pediatric behavioral/mental health within the context of natural/biological disasters at all stages of the disaster cycle is paramount for reducing the considerable potential psychological toll of such events. Previous research on pandemics shows that infectious disease outbreaks are often associated with increases in anxiety, depression, posttraumatic stress disorder (PTSD), substance use disorders, domestic violence, and child maltreatment [2]. The COVID-19 pandemic was no exception as there were numerous negative effects of the pandemic on children’s mental health. Children experienced trauma related to exposure to domestic violence, child maltreatment, separation from a hospitalized caregiver, and the loss of a family member. Additionally, children experienced isolation, depression, and anxiety related to either contracting the virus or concerns about contracting the virus, lockdown, changes in routine, and threats to safety and financial instability in the home. Children who live in poverty or rural/remote communities, identified as disenfranchised minorities, and have neurodevelopmental disabilities and other special health care needs are particularly vulnerable during pandemics and other types of disasters [3].

## 2. Ongoing Pediatric Behavioral/Mental Health Crisis

As the COVID-19 pandemic worsened child mental health, it also intensified the disparities and challenges with accessing care. In response to this, the American Academy of Pediatrics (AAP), the American Academy of Child and Adolescent Psychiatry (AACAP), and the Children’s Hospital Association (CHA) declared a national state of emergency in children’s mental health [4]. Prior to the pandemic, 1 in 5 children had a mental health disorder, with only 20% receiving care from a mental health provider [5]. Rates of childhood mental health problems and suicide rose steadily between 2010 and 2020, with suicide being the second leading cause of death in youth aged between 10 and 24 [4]. In comparison to 2019, between March 2020 and October 2020, mental-health-related emergency department visits increased by 24% for children aged 5 to 11 and by 31% for youth aged 12 to 17 [6]. Moreover, more than 140,000 children in the United States lost a primary and/or secondary caregiver due to COVID-19-related death, with youth of color being disproportionately affected.

As a reaction to the escalating pediatric mental health crisis just prior to the start of the COVID-19 pandemic, there was a national call to develop coordinated behavioral health disaster care capability, including response to natural/biological disasters such as endemics and pandemics, and to enhance situational awareness. To address regional and national need, the Administration for Strategic Preparedness and Response (ASPR) funded two Centers of Excellence, namely, the Eastern Great Lakes Pediatric Consortium for Disaster Response (EGL-PCDR) and the Western Regional Alliance for Pediatric Emergency Management (WRAP-EM), which both launched in 2019. In 2021, the Health Resources and Services Administration (HRSA) funded the Pediatric Pandemic Network (PPN) whose mission is “to empower health-care systems and communities across the nation to provide high-quality, equitable care to children every day and in crisis” [7]. The focus of this commentary is on the authors’ involvement in the EGL-PCDR (now Region V for Kids encompassing Ohio, Michigan, Illinois, Indiana, Minnesota, and Wisconsin) as co-chairs of the Behavioral Health Domain and as Behavioral Health Domain co-leads for the PPN.

Behavioral health clinicians are poised to build partnerships with key community partners, first responders, emergency department clinicians, and those working within the medical home and schools to lead outreach, education, and training in disaster preparedness and response. The intention of this commentary is to serve as an exemplar for taking a behavioral-health-informed approach to COVID-19 and to emphasize considerations for this type of approach for future pandemics/endemics. Even though the experience of the authors was through U.S.-funded programs, international organizations were also instrumental in responding to pediatric behavioral health needs during the COVID-19 pandemic, including UNICEF (e.g., https://www.unicef.org/reports/state-worlds-children-2021 (accessed on 21 May 2023)). UNICEF played a critical role in addressing inequalities related to access to vaccines and personal protective equipment, strengthening health care systems, and supporting children and their families. UNICEF made globally available psychosocial material on self-care tips for parents, positive parenting strategies, and strategies for promoting learning and play.

## 3. Role of Psychologists and Other Behavioral Health Professionals in the Response Phase

Psychologists and other behavioral/mental health providers can play a vital role in response to public health and medical crises. These providers can direct and assist with determining predictors of disease, risk and protective factors, and influences of disease course and patient recovery. Behavioral/mental health providers can also provide the needed support to employees experiencing secondary traumatic stress or vicarious trauma related to caring for critically ill patients [8]. In response to the pandemic, the authors of this commentary were involved in interdisciplinary collaborations that rapidly resulted in the development of products. One such product created in the first summer of the pandemic was the “Pediatric Exit Care Instructions for Confirmed or Suspected COVID-19”, designed for children and adolescents discharged from urgent care, the emergency room, or hospital and going into home isolation. The psychosocial discharge instructions were available nationally for download and customization by individual emergency department health care providers and facilitators. Another product that developed out of collaboration with infectious disease experts was a healthy restart playbook for supporting children to return to the in-person school setting. A team of academic medical center-based experts reviewed this playbook with school administrators and teachers via virtual consultation sessions, providing opportunities for questions and answers as well as open discussion. During these sessions, psychologists played a primary role in advising school personnel regarding how to encourage students to wear masks and engage in other safe behaviors. These two specific products highlight the need for quick improvisation in the mitigation and response phases of natural or biological disasters such as pandemics to minimize their psychological impact on children and their families.

Also in response to the COVID-19 pandemic, the authors’ use of dissemination of targeted strategies to a broad audience promoted positive psychosocial adjustment and resilience. This took the form of webinars provided to behavioral health clinicians, emergency department physicians, emergency management service providers, and psychologists and trainees through state psychological associations as well as media engagements targeting children and their families (i.e., television, internet, and blogs for APA-supported organizations and creation of psychosocial adjustment and resilience handouts). These handouts addressed several topics, including response phase strategies for parenting children with neurodevelopmental disorders and infants and toddlers during the pandemic, coping strategies specifically for adolescents, ideas for managing family stress, and expert guidance for obtaining optimal sleep for adolescents. Further details regarding these products and other efforts of the authors and members of the ASPR-EGL Behavioral Health Committee are in a white paper [9].

An inclusive approach considering special populations is vital during a pandemic. The authors of the commentary placed specific focus on children with special health care needs and children at high risk for maltreatment. Toward this end, a multi-institutional collaboration resulted in the development of a telehealth protocol for the evidence-based group parenting program ACT Raising Safe Kids (ACT-RSK). ACT-RSK is a primary violence prevention and intervention program developed by the American Psychological Association (APA) that focuses on developing and promoting positive parenting, decreasing the risk for child maltreatment, and fostering safe communities [10]. Parents’ nurturing and discipline practices in addition to parenting knowledge of key aspects of the ACT-RSK program (e.g., monitoring exposure to media violence) was evaluated pre- and post-intervention. Qualitative data were also obtained from both caregivers and ACT-RSK facilitators on the feasibility and acceptability of the program. The results of the study indicated that the pivot to virtual delivery of the model was feasible and acceptable by both groups and that negative/harsh parenting practices decreased (manuscript under review). This protocol was implemented, evaluated, and disseminated nationally and internationally to ACT-RSK trained providers. This pilot project, as well as telehealth delivery of behavioral health care more globally, not only mitigated the spread of COVID-19 but also connected patients in rural settings and the historically disenfranchised with the needed mental health care services while eliminating barriers related to transportation and childcare.

To further understand the impact of the pandemic on young children in childcare settings, media use, and sleep during the pandemic, one of the commentary authors (CIL) was a co-guest editor of a special issue of *Children’s Health Care* on pediatric aspects of the COVID-19 pandemic [11]. Both authors of the present commentary collaborated on research studies and/or a narrative review with psychologists or medical providers from other disciplines (e.g., developmental behavioral pediatrics and sleep medicine) within their own institution and at other institutions. In a study of parents/caregivers (primarily female) of 114 children aged 3 months to 10 years accessing Ohio childcare early in the COVID-19 pandemic, caregivers completed a survey to assess their pandemic-related stress as well as their general psychological distress, coping strategies, reported quality of caregiver–child interaction, and changes in child behavior/development. Caregivers reported their COVID-19-related stress as being 70.83 (1–100 from no stress to extreme stress), and 79% reported being moderately or extremely worried about COVID-19. Multivariable adjusted regression models were tested to determine the associations between pandemic-related stress and changes in aggressive behavior and social skills. Findings were that higher caregiver-reported COVID-19-related stress was associated with increased odds of worsening child aggression and social skills. These associations were partially mediated by the caregiver working from home and having difficulty with emotional regulation. This research highlights the need for monitoring children of caregivers experiencing higher pandemic-related stress [12].

Additionally, one of the commentary authors (KB) worked with public health experts and epidemiologists on active surveillance of the COVID-19 pandemic in childcare settings within and across a midwestern state. Passive surveillance data obtained from the state-operated incident reporting system and active surveillance data with self-administered reverse transcriptase polymerase chain reaction (RT-PCR) tests. Focus groups were also conducted to discuss mitigation strategies used by administrators/staff and recommendations for future practice to keep childcare centers open during a pandemic/endemic. Suggested mitigation strategies included increasing the financial capacity of childcare settings to purchase personal protective equipment and maintain appropriate staffing. The findings align with other studies conducted in the U.S. and in Europe, which indicated that childcare settings did not present elevated risk to the staff, children, or community when mitigation strategies were in place. This study has important implications as it helps to support childcare settings remaining open, which allows caregivers to work, promoting financial stability and opportunities for pre-academic learning and socialization [13].

Another study considered sleep and media use among 24 children aged 5–12 years with autism spectrum disorder (ASD) compared to 51 children with attention-deficit/hyperactivity disorder (ADHD) or other neurodevelopmental disabilities during the early stages of the COVID-19 pandemic (November 2020 to May 2021) [14]. Sleep information was collected via parents’ reports on children’s typical sleep duration, regularity, and disturbance/impairment. Predictors were demographics, group membership (ASD versus other neurodevelopmental disorders without ASD), electronic media use duration, and COVID-19-related distress. Findings were that media use duration was not significantly related to any of the sleep outcomes. Unexpectedly, in a subgroup analysis that included participants who completed a COVID-19 exposure and distress measure, greater media use related to less sleep-related impairment and disturbance. This study demonstrates the importance of carefully tailoring sleep hygiene recommendations for these at-risk children, particularly in the midst of a disaster. Finally, a narrative review of sleep research was conducted by searching for papers published between March 2020 and June 2021 during the pandemic [15]. The papers were organized by child developmental period and revealed high rates of sleep disturbance along with risk and protective factors. This review contained real-life clinical case examples from one of the commentary’s authors (CIL) of the unique sleep challenges faced by children and adolescents during this pandemic. Overall, preschool-age children went to bed later and were getting less sleep; school-age children felt greater anxiety at bedtime, more than two nighttime awakenings, nightmares, and daytime sleepiness; and adolescents had a delayed sleep schedule and difficulty initiating and maintaining sleep but experienced less daytime sleepiness. Results of all of these studies may be used to inform recommendations in real time and to prepare for future endemics/pandemics.

## 4. Learning from Response and Recovery Phases to Inform the Preparedness Phase

What we learn in the response and recovery phases helps to inform steps for the preparedness phase. Efforts in each of these phases are therefore not mutually exclusive. One critical step is to encourage hospital systems to complete an environmental scan in the emergency department to determine the capacity and capability of a mental health surge, which is commonly associated with endemics/pandemics. In addition to mandates to screen for trauma and suicide risk in the emergency department (with instruments such as the Screening Tool for Early Predictors of PTSD (STEPP), Ask Suicide Screening Questions (ASQ), and the Columbia Suicide Severity Rating Scale (C-SSRS)), development of hospital-specific protocols is vital to connect with mental health providers, particularly those who can provide teleconsultation. In line with this, we recommend identification of a pediatric mental health champion at every hospital/organization. The pediatric mental health champion should receive training in Psychological First Aid and Skills for Psychological Recovery. These training programs can be accessed through the National Child Traumatic Stress Network (NCTSN) website and are classified as evidence-informed modular approaches to help survivors manage post-disaster stress and adversity [16]. These are not formal mental health treatments and are approaches that a variety of helping professionals can employ to stabilize families, build problem-solving skills, promote positive activities and helpful thinking, and facilitate rebuilding healthy social connections. Therefore, we also recommend encouraging health care providers and other emergency department staff to obtain training in Psychological First Aid. Furthermore, interdisciplinary tabletop exercises allow for practice of surge protocols to simulate how to triage patients based on the number and type of exposures and to demonstrate the use of telehealth in real time to leverage available resources.

During a pandemic, it is imperative to keep the patient volume of the emergency department down so that focus can remain on the treatment of injuries and/or disease. In preparation, we have led efforts to identify and address mental health concerns at various points along the health care continuum. One way to do this is through platforms such as ProjectECHO [17]. ECHO stands for Extension in Community Healthcare Outcomes, and this model encourages participants to present real, anonymized cases to specialists for discussion and recommendations. This knowledge-sharing model offers a continuous loop of learning, mentoring, and peer support. As an example of this strategy to reduce the volume of children and adolescents seeking mental health assessment and triage in the emergency department, we utilized this platform with community-based pediatricians to increase their knowledge of empirically supported screening practices and management of depression within the context of the medical home.

## 5. Developing a Crisis Plan at Multiple Points of Care for Pediatric Behavioral Health

We recommend proactive development of a crisis plan at multiple points of care. This requires a paradigm shift from a “boots-on-the-ground only approach” to identifying how psychologists and other behavioral/mental health providers can flexibly put their skill sets into practice to diminish stress during a pandemic and promote well-being [18]. Additionally, psychologists can use their research training as social scientists to inform practice. A call to action for behavioral health workforce development in the arena of pediatric disaster preparedness and response is needed. Psychologists and other behavioral/mental health professionals can use their training in Psychological First Aid, active listening, validation of emotional responses, teaching coping skills, and ability to provide reflective supervision to support first responders and patients. Moreover, many of these providers can promote taking a public health approach centered on prevention and intervention at the community level. These initiatives include psychoeducation, empowerment programs, strengthening support networks, and leveraging community leadership to promote and disseminate psychosocial prevention strategies. There are five strategies proposed to address the mental health impact of COVID-19 on communities. These include (1) engaging and partnering with community leaders; (2) establishing support networks, such as self-help groups to promote psychological resilience; (3) providing community mental health outreach, education, and training (e.g., Psychological First Aid, Mental Health First Aid, and the Community Resilience Model); (4) working in tandem with community health workers; and (5) focusing on community empowerment. The latter is important because studies have shown that a greater sense of community serves as a strong predictor of lower levels of depression and improved physical health [2].

## 6. Lessons Learned Related to Pediatric Mental Health during the COVID-19 Pandemic

The National Advisory Committee on Children and Disasters (NACCD) identified lessons learned from the COVID-19 pandemic as they relate to the ongoing mental health crisis. The recommendations to HHS include developing “a children’s disaster mental health working group to support emergent and urgent mental health services for children, identification of funding mechanisms for disaster behavioral healthcare where and when local/regional resources are exhausted, pre-disaster training and just-in-time training for those who routinely care for children, grants for disaster mental health training for clinicians and non-clinical professionals in the health system, and proactive funding for behavioral health recovery interventions in schools.” [3]. The HRSA-funded Pediatric Mental Health Care Access (PMHCA) program in part addresses these recommendations. The PMHCA program provides pediatric mental health care technical assistance to emergency departments across the country with the goal of developing and disseminating best practices to community-based hospitals through a hub-and-spoke model. The authors of this commentary are in the process of identifying teleconsultation best practices supported through the Emergency Medical Services (EMS) for Children and Improvement Center (EIIC). Additionally, next steps related to behavioral health for the PPN include educating first responders to work with children with special health care needs, creating a national registry of clinicians who provide trauma-informed intervention as defined by disaster mental health criteria, adapting mental health toolkits for children and their families, identifying a coordinated response to those presenting with suicidality to the emergency department, increasing collaboration with schools, and incorporating behavioral sleep medicine within all levels of trauma-informed intervention.

## 7. Psychologists and Other Behavioral Health Providers Moving toward the Recovery Phase

At the time of writing this commentary, we continue to be in the response phase of the disaster cycle related to the COVID-19 pandemic and are moving toward a focus on recovery. Based on our experience, efforts in the recovery phase should concentrate on increasing and maintaining behavioral health competency through identification, development, and delivery of training programs for workforce development. The authors are in the process of creating a pediatric behavioral health preparedness and response curriculum through their work as co-leads of the Behavioral Health Domain of the PPN. This will allow additional behavioral health clinicians to become disaster ready. The curriculum will include education on the disaster management cycle; core components of creating a trauma-informed organization, including identification of instruments that assess for ACEs, posttraumatic stress, and suicidality; and strategies for taking a population-based approach to mental health. Guidelines within the curriculum will also encompass tips for conducting interdisciplinary research, seminal research articles, links to family readiness toolkits, and recommendations for anticipatory guidance related to preparedness. Telehealth practice rules and strategies to optimize teleconsultation will also be available as part of the curriculum. Insurance coverage of telehealth has provided the opportunity for increased access for historically marginalized populations.

Of utmost importance is encouraging other licensed mental health providers to seek additional specialty training in empirically informed/evidence-based interventions for responding to the immediate aftermath of a disaster and treating associated trauma symptoms (such as insomnia and nightmares) and grief. These include trauma-focused cognitive behavioral therapy (TF-CBT), trauma and grief component therapy for adolescents (TGCTA), cognitive behavioral therapy for insomnia (CBT-I), and imagery rehearsal therapy (IRT) for nightmares. The core components of TF-CBT are psychoeducation, parenting skills, relaxation, affect modulation and regulation, creation of a trauma narrative, cognitive processing of the narrative, exposure to the narrative, and strategies for enhancing safety [19]. TGCTA is a manualized group or individual treatment program for older children and adolescents who have been exposed to trauma or are traumatically bereaved, such as by losing a parent or caregiver during a pandemic [20]. This program addresses a broad range of clinical problems, such as coping with reactions to distress, managing reminders of trauma or loss, and addressing the interplay between post-traumatic stress and grief reactions. CBT-I is an empirically supported intervention to address one of the primary symptoms associated with trauma and PTSD—insomnia—and has received considerable research support for its use with children and adolescents [21]. CBT-I includes first-line strategies of stimulus control, sleep restriction, and sleep hygiene. Stimulus control relates to the connection of the brain with the bed as a place for sleeping with the goal of 85–90% sleep efficiency (time in bed/total sleep time). Sleep restriction involves limiting the opportunity for sleep to match the child’s or adolescent’s biological need for sleep, which varies by age, genetics, neurodevelopmental condition, etc. Finally, sleep hygiene includes many factors, such as building up the need for sleep through daily moderate-to-vigorous physical activity, limiting naps, and maintaining a regular circadian rhythm by avoiding lengthy weekend morning sleep-ins. Second-line strategies involve relaxation training and cognitive restructuring relating to thoughts about sleep and treatment of insomnia symptoms. IRT is a therapeutic method for addressing bad dreams or nightmares and involves encouraging the child to devise a new dream they would rather have or to change an aspect of a recurring dream to become more positive. This method for children requires practicing the new dream daily for 10–15 min via drawing and/or talking about the preferred dream with parents or caregivers. This dream will then replace the bad dream. Studies of IRT in children have yielded promising findings of reducing nightmare frequency and emotional intensity [22,23]. With regard to these specialized therapeutic approaches, psychologists and other licensed mental health care providers becoming proficient in one or more of these is crucial for behavioral health workforce development in pediatric disaster preparedness and response efforts.

## 8. Conclusions

Through all of the phases of a natural/biological disaster such as a pandemic, strategies for everyday readiness to tailoring and developing pediatric behavioral health materials and collaborating with medical providers and community partners should be applied. Psychologists and other behavioral health providers play a vital role in the psychological well-being of children and their families. Moving away from a boots-on-the-ground solely approach and embracing the available technology of telehealth and teleconsultation allows behavioral health professionals from many different specialties to join the ranks of those prepared to respond in the event of a disaster. We hope that this commentary can serve as a roadmap for our behavioral health colleagues to identify needs within their own community, establish a wide network of partnerships, and seek out additional disaster preparedness and response information and specialized training. This will ensure that children and their families are adequately supported through all the phases of the disaster management cycle, from mitigation/risk reduction of potential psychological consequences, to preparedness via specialized training, to response in real time in conjunction with partners using novel delivery methods (e.g., telehealth), and finally to recovery to pre-disaster levels of functioning.
